# Sudden Cardiac Death As a Result of Neglected Hypopituitarism

**DOI:** 10.5812/ijem.5374

**Published:** 2013-04-01

**Authors:** Farhad Hajsheikholeslami, Shahrooz Yazdani

**Affiliations:** 1 Prevention of Metabolic Disorders Research Center, Research Institute for Endocrine Sciences, Shahid Beheshti University of Medical Sciences Tehran, IR Iran; 2 Department of Cardiology, Taleghani Hospital, Shahid Beheshti University of Medical Sciences Tehran, IR Iran

**Keywords:** Hypopituitarism, Electrocardiographic Changes, QT prolongation, Polymorphic ventricular Tachycardia

## Abstract

Cardiac involvement infrequently occurs in hypopituitarism, and lethal cardiac arrhythmias are rarely reported. We present a middle age female who died as a consequence of refractory ventricular arrhythmia whose medical history and previous laboratory investigation were consistent with hypopituitarism. We conclude that hypopituitarism may lead to electrocardiographic changes and malignant ventricular arrhythmia and should be included in laboratory investigation and differential diagnosis of patients presenting with long QT syndrome

## 1. Introduction

Hypopituitarism is considered when a defect in some of the anterior pituitary axes results in failure of one or more target gland. The insult to the pituitary and hypothalamic area may be acute or chronic hence the clinical presentation may vary accordingly and in the majority of cases, it intervenes with a normal quality of life and may decrease life expectancy secondary to atherosclerotic complications or cerebrovascular accidents (1). Among various presentations of hypopituitarism, lethal ventricular arrhythmias are very rare. Here, we retrospectively present a neglected case of hypopituitarism, which presented with the sudden onset of refractory ventricular arrhythmia unresponsive to resuscitative effort.

## 2. Case

**Figure 1.**
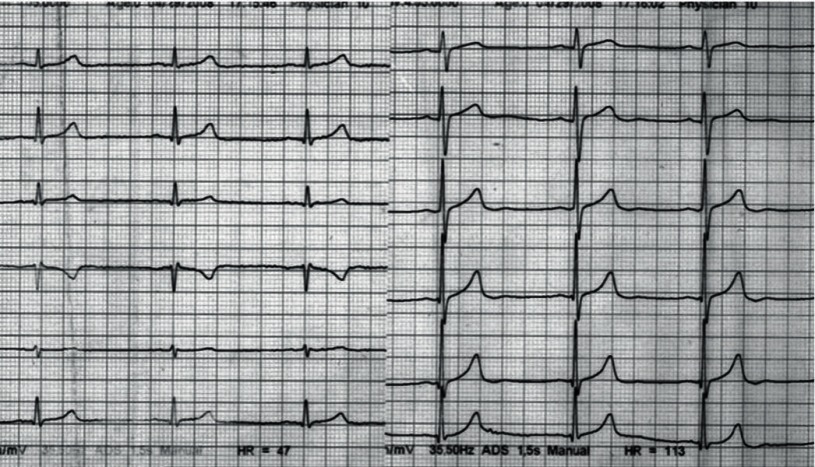
12 Leads EKG at 29.4.2008 Showing Sinus Bradycardia

**Figure 2.**
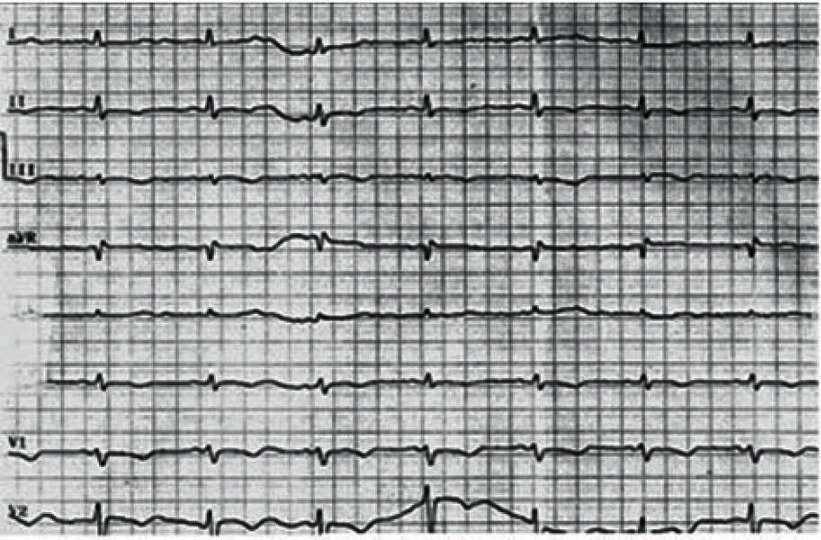
12 Leads EKG at Hospital Admission on 27.10.2009 Sinus Bradycardia and QT Prolongation

**Figure 3.**
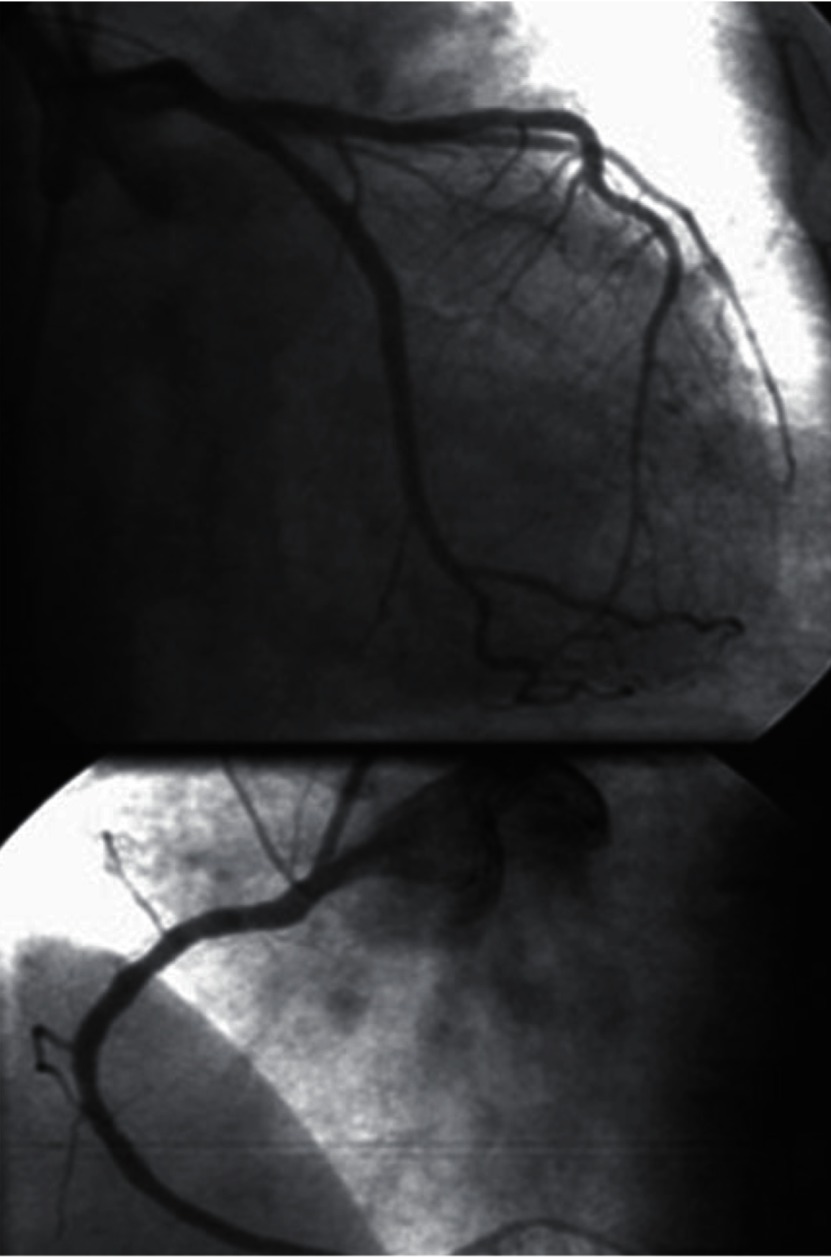
Coronary Angiograms Showing Minimal Intimal Irregularities and No Evidence of Significant Stenosis

A 58 year old female lost consciousness in her room while preparing for a wedding ceremony and was instantly brought to the emergency room in 28.10.2010.Her cardiac monitoring on arrival showed polymorphic ventricular tachycardia degenerating to ventricular fibrillation, which was unresponsive to prolonged CPR efforts, and she died one hour after arrival to ER(emergency room. Examinations including electrolytes and blood sugar was reported to be normal, retrospective questioning of close family members and reviewing the previous medical data revealed that during last 36 months she had frequent visit to her family physician complaining of weakness and vertigo, her work up on 12 March 2007, while taking Levothyroxin100 ug daily and occasional 5 mg Prednisolone tab (Table 1) revealed hypothyroidism and decreased fasting cortisol level, her ECG (Figure 1) on 27.10.2009 time of admission to another hospital for severe fatigue and atypical chest discomfort uncovered sinus bradycardia and prolonged corrected QT interval QTc 600 msec (Figure 2), during hospitalization selective coronary angiography and left heart catheterization was performed and revealed normal epicardial coronaries Figure 3, and left ventricular function. Echocardiography at the same admission was reported normal. Unfortunately, no other imaging studies (e.g. brain MRI) were available in this case hence no clear cause for hypopituitarism could be elucidated.

**Table 1.** Laboratory Findings

TEST	Patient	Normal Range
**T3**	0.5 ng/mL	0.4-1.8
**T4**	1.2 ug/dL	4.5-13
**TSH**	4.5 mI u/L	0.17-8.9
**FSH**	1.7 mIU/mL	> 20
**LH**	0.5ml Iu/mL	2.1-17.7
**Prolactin (EIA)**	2.2ng/mL	2.1-17.7
**Morning cortisol (EIA)**	0.1ug/dL	5-25
**Ferritin**	658 ng	10-120
**FBS**	71 mg/dL	70-110
**Cr**	1.2 mg/dL	0.6-1.2
**Uric acid**	5.1 mg/dL	2.5-6
**Cholesterol**	403 mg	< 200
**TG**	348	< 150
**HDL**	40	35-90
**LDL**	293	< 130
**Hb**	12.7 g	
**Hct**	37%	
**Platelets**	87000	140-400K
**ESR**	20	
**CPK**	1409 IU/L	25-225
**CPK-MB**	44	0-24
**Troponins T**	< 0.01 ng/mL	< 0.03 ng/mL

## 3. Discussion

Hypopituitarism was first described in 1914 by the German physician Dr Morris Simmonds (1).

since then an enormous growth of knowledge about the pathophysiologic and management of this condition have been developed but the vague clinical presentation is still an obstacle to timely diagnosis and proper management which sometimes leads to extreme and unusual morbid presentations as seen in our case.

Sudden unexpected death (2) and Cardiovascular complications of hypopituitarism have been described previously but reports of lethal cardiac arrhythmia are very rare (3-5) as are electrocardiographic changes in hypopituitarism (6, 7) the electrocardiographic changes that are considered to be associated with hypopituitarism are giant T inversion, QT prolongation and ST changes. The exact cause of electrocardiographic changes in such cases is not defined but hypoglycemia, catecholamine surge secondary to hypoglycemia and hypomagnesemia, or decrease ACTH level (2) are considered as possible cause, such changes mimics ischemia and intracranial pathology but in our patient coronary disease was ruled out by coronary angiography, other possible causes of QT prolongation such as genetic or drug induced may be considered in our case but history was negative for use of such medications and as seen in (Figure 1) the ECG in 29.4.2008 QT interval was normal before possible aggravation of endocrine condition which may have been triggered by discontinuation of drugs or emotional stress before onset of ventricular arrhythmia .

## 4. Conclusions

We described a case presented with refractory ventricular tachycardia unresponsive to medical therapy with subsequent laboratory finding in favor of neglected panhypopituitarism. It is concluded that cardiac arrhythmia as a rare presentation of neuroendocrine disorders should be considered in differential diagnosis of long QT syndrome and ventricular arrhythmia.
